# We Are Displaced, But We Are More Than That: Using Anarchist Principles to Materialize Capitalism’s Cracks at Sites of Contemporary Forced Displacement in Europe

**DOI:** 10.1007/s10761-023-00696-5

**Published:** 2023-02-07

**Authors:** Rachael Kiddey

**Affiliations:** Department of Archaeology, University of Cambridge, Downing Street, Cambridge, CB2 3DZ UK

**Keywords:** Community archaeology, Displacement, Anarchism, Contemporary archaeology, Activism, Consensus

## Abstract

This article charts the development of The Made in Migration Collective, a coalition of displaced people, academics, and creative professionals that was developed during a recently completed British Academy postdoctoral fellowship. Following discussion of how archaeology and heritage are under attack globally from far-right nationalism and specifically within the UK, I provide examples of how community archaeology can highlight fissures in capitalism. I follow others in interpreting anarchism as a potential form of care. Two public heritage exhibitions – one digital, one “live”—which were collaboratively produced by The Made in Migration Collective are reflected upon.

## Introduction

*“Anarchism is the heart of the movement, its soul; the source of most of what’s new and hopeful about it.”* (Graeber 2002:62)

It is a cold evening in late December 2019. I’ve just boarded a train at Syntagma station headed for Athens International Airport. With my rucksack gripped firmly between my knees and my passport and money stashed in an inside pocket, I lean my head against the train window. Outside, Syntagma station speeds up until all I perceive is a cinematic blur of graffiti and concrete, power cables etch an inky scrawl against a moonlit sky. I am heading home after undertaking fieldwork for my British Academy funded postdoctoral fellowship, “Migrant Materialities,” in squats, on the street, and in volunteer-led support spaces, in collaboration with displaced people. I feel tremendous guilt that I can “go home” while my collaborators must continue to endure permanent, often dangerous, uncertainty. The “scream” (Holloway 2010) inside of me is so overwhelming I fear it might spill out as a howl. I “care”; others “care.” Capitalism does not “care” about anything but economic growth at all costs (cf. Flexner 2020). In this paper, I use theories and praxis drawn from anarchism and intersectional ecofeminism to contextualize ethnographic and contemporary archaeological fieldwork undertaken collaboratively with people living in various situations of forced displacement in three European countries – Greece, the UK, and Sweden. Fieldwork was conducted between April 2018 and January 2020 although relationships with displaced people were, in some cases, first established in early 2017 during a previous research project. Since fieldwork ended, the Collective has co-produced two public heritage exhibitions, upon which I reflect at the end of this article.

As a researcher, I have always prioritized the social relations – human relationships – involved in studying material and visual culture with marginalized people. I “start from the particular, not the totality” (Holloway 2010:20, also cited by Wurst and Dézsi, this volume) where the sites and assemblages involved are invariably defined by their misfitting – space turned place through its use by homeless or displaced people (cf. Kiddey, 2017a, b, 2019). Such sites are characterized by types of mutual solidarity, out of necessity if not always ideology. The sites and assemblages discussed in this article are all either intentionally or accidentally cracks in capitalism’s façade.

By way of theoretical and political context, I first provide a brief overview of some of the ways in which anti-establishment narratives of the past are under attack globally from far-right nationalism, where, specifically within the UK, sustained attack is promulgated by the current Conservative government. Following Borck (2018), I contend that theory and praxis drawn from anarchism can be useful in countering such attacks, enabling interpretations of the past in which “future histories'' could be more egalitarian. I contend that anarchist derived approaches to social organization are particularly powerful when coupled with intersectional ecofeminism where care is prioritized. I am specifically drawn to ecofeminism because it can be conceived as *both* a theory and a movement (Estévez-Saá and Lorenzo-Modia 2018). By connecting feminist notions of care – care for one another, care for material and visual culture which testify to “alternative” ways to know the world, and care for the future – with concerns for how women and other human and non-humans continue to be oppressed by discriminatory attitudes and policies, we stand a better chance of rebalancing our relationships to the world in ways which do not prioitize economic growth (for increasingly few humans) over, and at the expense of, absolutely everything else.

I then offer a necessarily reduced discussion of how anarchist approaches can be useful to community archaeology, focusing on how prefigurative praxis was used to co-develop *The Made in Migration Collective*. I outline how using consensus decision making principles to co-design the research and decide the direction of the project led to unorthodox but fruitful methods of engagement and surprising and creative outcomes. The aim of this article is to provide examples of how such methods of collaboration in community archaeology can greatly help to highlight fissures in capitalism and assist in the expansion and connection of our “network of voices” more effectively (Subcomandante Marcos, cited in Graeber 2002:63).

In some ways, displaced people are forced to embody “cracks” in capitalism because they are routinely excluded from the “mainstream,” prevented from involvement in the reproduction of the capitalist mode. Similar to the “unhoused” people with whom Aaron Howe works (Howe, this volume), displaced people often have no option but to engage in “useful labor” in order that they might survive. Correspondingly, many of the temporary camps and flats managed by international humanitarian organizations and private companies instructed by the state rely on “abstract labor,” which function to reproduce capitalist value. Indeed, the history of the humanitarian sector is itself a story of the creation of a multi-billion-dollar industry (Barnett 2013). The global Covid-19 pandemic exposed inequalities which many of us have long argued existed so that even the most ardent capitalist struggled to deny them…for a short while. The inequalities and injustices faced by many members of *The Made in Migration Collective* are greatly exacerbated versions of those faced by all who find ourselves – and our non-human fellows—in the grip of rabid global capitalism, its cruelties, and wanton destruction.

I go back to that moment at Syntagma as I boarded the train to the airport, the “scream” lodged in my throat, a painful combination of absolute fury and near total despair – that it is wholly unethical to idly give in to capitalism. It is not “just the way the world is” that some people have the means to cross a border, to fill their children’s bellies with food and their brains with education, while others are condemned to starve and suffer. It is the result of a system of relations (capitalism) which is predicated on inequality, supported by reactionary populism and the hardening of national borders (cf. González-Ruibal 2018a; McAtackney and McGuire 2020). People do not flee across borders, leaving behind everyone and everything they have ever known for an easy life! They do it because their homelands are rendered uninhabitable through war and intolerable economic conditions which are often the violent legacies of colonialism, from both of which “my” nation state (the UK) continues to directly profit, through its ongoing imperial grip on vast swathes of the planet and its unenviable position as the world’s second largest arms dealer. Humanity – if not nation states—can do much better. Archaeology and anthropology can provide varied evidence for how, if we engage principles drawn from anarchism and organize our diverse networks.

## The “War on Woke”: Cultural Symptoms of the Global Shift to the Right

Archaeologists and heritage scholars have been forthright in critiquing how material culture is embroiled in capitalism (Zorzin 2015) and articulating the unsettling ways in which heritage, nationalism, and conflict are related (Meskell 2018). There has never been a time in my professional life as an archaeologist when narratives which seek to expose injustices of the past and their ongoing violence have been under more direct threat from the state than right now (cf. Hamilakis 2018:518). In the UK, the slow but sure shift to the political right took a giant leap further right in 2016 when the voting public chose, by a very narrow margin, to leave the European Union in the Brexit referendum. Across Europe, in Austria, Switzerland, Hungary, The Netherlands, Sweden, Denmark, Finland, Greece, Italy, Spain, Belgium, and other countries, support for far-right nationalist parties has risen to almost half of the vote in some cases. Events in other parts of the world show that this alarming problem is not confined to Europe. In the US, Donald Trump’s presidential election was followed by four years of policy and in/action that served to dramatically undermine previous efforts to improve social equality and environmental stability, and it ended with surreal scenes of far-right supporters storming the Capitol, encouraged to do so by the then President of the United States! In other parts of the world, the pattern is depressingly similar – Bolsonaro in Brazil, Piñero in Chile, Erdogan in Turkey, Narendra Mohdi in India. Neoliberalist economics and nationalist identity politics are a “marriage of convenience” (Ehmsen and Albert 2018:2) that violently defines and hardens political borders and aggressively ascribes and polices identity—you’re either in or you’re out (Holloway 2010:72) – and that often depends on things a person cannot change, such as their skin color, sexuality, or the place of their birth. Tolia-Kelly (2019:591) notes that:

diasporised identities are disassembled and reassembled to form a ‘weave of differences’ that refuse ‘authenticity’, ‘tradition’ and as such narratives of ‘origin’ connected to blood and soil. The 21st-century resurgence of nationalism is a response to an imagined threat of syncretic creolisation. Although, rather than being experienced at the territory of the colony, the conditions are experienced at the centre of imperial thought and cultural dominance, Europe.

As a UK based community archaeologist working with displaced people living in Europe, the type of work that I do is exactly the kind of heritage that the UK’s former Culture Secretary, Oliver Dowden, wants to dismiss with his “war on woke” (Guardian 2021). What Dowden and the current right-wing UK government are doing is launching an egregious attack on interpretations of the past which explicitly aim to offer counternarratives to their preferred historical discourse, which is capitalist, elitist, iniquitous, jingoistic, and unrepentant of atrocities such as the Atlantic slave trade. The current UK government is correct not to underestimate the important roles played by heritage, historic monuments, and archaeological artifacts in maintaining their façade, and they are scared by its potential. In April 2022, the Police, Crime, Sentencing and Courts Bill received royal assent and passed into UK legislation. It means that people who damage statues face up to ten years in prison (statues such as that C word fellow who was pushed into Bristol harbor during a Black Lives Matter protest in the summer of 2020) (Grey 2020). This means that UK statue-wreckers could receive five years more prison time than rapists (Everard 2021). We might view Black Lives Matter supporter reactions to statues of colonialists and slavers, such as the aforementioned Bristol case, “as a reminder of white reluctance to critically revisit the still-persisting effects of past colonialism in our societies, and…the emotional damage that this inaction causes” (Colomer 2020:3). Or, as Lewis Borck (2018:229) puts it:

archaeological sites are being mobilized not just to legitimize the state, but to create a future history where alternative power structures – egalitarian, non-state, Indigenous, pre-colonial – are seen as impossible to achieve; or worse, are forgotten.

Several articles in this volume contend with the effects of the historical present. For example, in his autoethnographic article, Oswaldo Hugo Benavides discusses the *enchaquirados* and the transgender community living in Engabao (a small fishing village in Ecuador). Benavides assesses how “alternative ancestral modes” of living continue to survive despite larger political forces, such as the censorship of Indigenous resistance by mainstream media companies, and other aspects of the heavy threat from capitalism (Benavides, this volume).

As archaeologists, we are familiar with studying the construction and formation of human societies which predate capitalism and colonialism (Gonzalez-Tennant 2018; Flexner and Gonzalez-Tennant 2018:217). Where before we might have done this out of interest in the origins of humanity, now we must do it to prevent even greater global authoritarianism (Fisher 2009). University departments and cultural heritage institutions constantly require us to demonstrate the “impact” and “relevance” of our work – the “war on woke” is a battle that requires archaeologists and scholars of all past-facing disciplines to pick up their tools, not down them (cf. Holloway 2010:205–215).

## Anarchism: A Way to Protect Diversity of Future Archaeologies

As has been usefully discussed by archaeologists before, anarchism literally means “without leaders” (Borck and Sanger 2017) but it does not mean total chaos either. As David Graeber (2002:70) wrote, twenty years ago:

[Anarchism] is not opposed to organization. It is about creating new forms of organization. It is not lacking in ideology. Those new forms of organization *are* its ideology. It is about creating and enacting horizontal networks instead of top-down structures like states, parties, or corporations; networks based on principles of decentralized, non-hierarchical consensus democracy. (emphasis in original)

Although plenty of Indigenous groups traditionally use ethical practices which we might recognize to be non-hierarchical and based around consensus, the modern anarchist movement began in nineteenth-century Europe, led by figures such as Kropotkin, Proudhon, and Bakunin. The “anarchist century” (Gemie 1996:417) was a time of empire and rapid industrialization; the atrocious effects of colonialism and slavery were clearly visible, inequality, and literal Dickensian poverty abounded. These conditions led many working-class Europeans to seek a better life across the Atlantic, in both North and South America, such that by the 1880s, Europeans were exporting not only capitalism, disease, and racism but also, in some cases, anarchist principles. At this point (and putting aside not unimportant practical differences between communist and individualist anarchists for a moment [see Kinna 2020:42–49]), it is perhaps useful to acknowledge that the anarchist movement was also advanced by plenty of non-white, non-male anarchists. Examples include Louise Michel (France, 1830–1905), Lilian Wolfe (England, 1875–1974), Lucy Parsons (1851–1942, North America), Voltairine de Cleyre (1866–1912, North America), Emma Goldman (1869–1940, North America), and Federica Montseny (1905–94, Spain but of Catalan heritage)—to name a few. It is also right to note that anarchist theorists of the nineteenth and twentieth centuries were, at times, just as racist (Goldman and de Cleyre), misogynist (Bakunin), and likely to patronize Indigenous peoples (Kropotkin) as their non-anarchist contemporaries. However, while it is imperative that we continue to expose and confront these prejudices and interrogate what these mean for both oppressed people and anarchist philosophy today, anarchist thinking remains useful.

A commonly recognized problem is that anarchism is generally so maligned and misunderstood that even left of left-leaning people often fear it, conceiving of anarchism as the total breakdown of society, a riot between balaclava-clad thugs hurling Molotov cocktails. Anarchism is often described as “Marxism’s poorer cousin” (Graeber 2004:3) or preferred by “peace-disturbers, wild, violent ignoramuses, who were jealous of the successful in life and fit only for prison or an asylum” (de Cleyre 2005[1908]:53; see also, Kinna 2020:129–133). This pejorative characterization of and refusal to “respectfully engage” (Graeber 2002:62) with an entire political discourse of course suits those who currently benefit most from capitalism, those who want us to believe that “There Is No Alternative” (cf. Muhr 2010). Although there is not – can never be – one agreed way to be anarchist, I follow David Graeber (2013) in interpreting anarchism foremost as a form of care through the way it centers the fundamental right for people to freely live their best lives, relying on mutual aid and solidarity. Briefly, at the start of the Covid-19 pandemic in the UK, we witnessed the formation of thousands of volunteer-led community groups which sought to support one another by meeting vital community needs without relying on state organizations or corporations (Power and Benton 2021). Often defined as “mutual aid” groups, there was more than a tinge of anarchism about how such groups evolved through people *caring* for one another in a crisis (cf. d’Alpoim Guedes et al. 2021). Care is an attribute that capitalism has often dismissed and shoved in a box marked “Women’s Work” (Hester 2018). Perhaps one way to make anarchism more palatable then, to those who misunderstand it, is to focus a while on its traditionally feminized qualities and further highlight the myriad ways in which anarchism prioritizes “affective relations, sensuality, playing, laughing, loving” (Holloway 2010:16). If we truly are to imagine a future in which the world broadly and archaeology specifically have a sustainable future, we could do worse than embrace methods of collaboration which are drawn from anarchism – from its fundamental call to question authority in human societies to more recent related discourse on “degrowth” (Flexner 2020; see also Kallis 2018).

Arguably, if we take the full existence of *Homo Sapiens* as a species, the norm for most of humanity was forms of social and political organization which were non-state based (Kropotkin 2020 [1902]; see also, Kinna 2016). While not all forms of human social and political organization prior to the development of nation states were anarchist or always more egalitarian than we see in the world today (cf. Graeber and Wengrow 2018), nation states, supported by institutions designed to underpin them—the armed forces, the judiciary, the police, state-sponsored school—have colonized the vast majority of the world and entrenched global inequality through capitalist extraction, made possible by the threat of (or actual) violence. State sanctioned institutions are the only bodies “allowed” to threaten to or actually incarcerate and detain people (residential schools, detention centers, prisons), to routinely inflict physical violence (through police batons and torture), and to kill (through corporal punishment and war). To my mind, statism far exceeds anarchism as the more dangerous form of social and political organization. In some cultures, anarchism is less feared and has been a more readily recognized way of life for some considerable time. Take Africa, for example: “ideals underlying anarchism may not be so new in the African context. What is new is the concept of anarchism as a social movement… Anarchy as an abstraction may indeed be remote to Africans, but it is not at all unknown as a way of life” (Mbah and Igariwey 1997:27; see also, González-Ruibal 2014).

One of the arguments which persists for “there is no alternative to capitalism” is that the choice is binary – that we have to choose between capitalism or communism, and that existing global communist regimes reveal the problems of power just as explicitly, if not worse. Anarchists have been arguing this for years (Bakunin 2010 [1872]; de Cleyre 2005:60; Goldman 2012 [1910]). One of the ways in which we can answer this false dichotomy is by adopting the anarchist notion of prefiguration – that is, actively adopting the non-hierarchical models of social relations that we want to see in the world, and applying them to every aspect of our professional and personal lives (Borck 2018; Graeber 2013). Change comes from action, from the “doing” (cf. Maeckelbergh 2011). Archaeologists routinely study examples of how this has been done in the past, all over the world, and how it continues to thrive in cracks within capitalism (see Lekakis 2020 for useful discussion of cultural heritage in the realm of the commons). In the next section of this article, I introduce the background to my recently completed research project, “Migrant Materialities,” and describe the development of The Made in Migration Collective. 

## Background to “Migrant Materialities” and the Formation of “The Made in Migration Collective”

My postdoctoral research project was funded by the British Academy and titled, “Migrant Materialities” (2018–2022). Contributing to a growing field of anthropological archaeological research into contemporary human migrations (for example, Dé Leon 2015; Hamilakis 2016; Hicks and Mallett 2019; Soto 2016), the focus of my research was the material and visual culture of contemporary forced displacement in Europe. I set out to better understand how displaced people use and adapt materials to serve practical and emotional purposes, to sustain personal and cultural identity, autonomy, and well-being. My research ethos has always been that good ethnographic and archaeological work should be collaborative, participatory, public, open, and feminist (Kiddey 2020). To achieve this, I engage principles and praxis drawn broadly from anarchism for three main reasons. The first is a dislike of anything that is authoritarian or hierarchical and a personal commitment to disrupt such structures. The second is that I believe that new knowledge about human relationships with the material and natural world is best produced collaboratively. Third, following others, I consider attributes arising from anarchism – consensus, joy, conviviality, mutual aid, egalitarianism – to be “an ethic of care” (Kiddey 2017a, b: 52–69; see also, Flexner 2020:164).

By taking it “slow” (Caraher 2019) and making the time to prioritize the social relations involved in archaeological research—co-designing *all* aspects of a project—different forms of knowledge are more effectively “braided” together (Atalay 2012:27). Knowledge is produced in ways which are meaningful, potentially even transformative, for everyone involved (Kiddey 2017b; see also Betasamosake Simpson 2017). By organizing community archaeology projects according to broadly anarchist principles, they can become a form of “joyful militancy” (bergman and Nick 2017:14–15). By taking our *time* about things, we also reclaim *space* (cf. Lukàcs 1971), and thus stride towards, “exposing, delegitimizing and dismantling mechanisms of rule, while winning ever larger spaces of autonomy” (Graeber 2002:68).

In 2014, as my doctoral study of contemporary homelessness in two English cities drew to a close (Kiddey 2017a, b), I came to meet more and more people, on the street and in squats, who were “failed” asylum-seekers. Or more correctly, people whose asylum claims had been rejected by the UK government and had become criminalized through what Monish Bhatia (2020) wryly terms “crimmigration,” and thus had no further recourse to welfare or housing assistance. Some people whose claims are rejected are deported back to countries still at war or run by regimes which will likely cause them serious harm (often death), as punishment for previous political or humanitarian activities or due to prejudice over sexuality or gender identity, for example. A few of those whose asylum claims are rejected evade deportation but they do so with their only choice then being to exist on the margins of so-called “mainstream society,” with no right to work or state protection from exploitation. This is how, often traumatized and extremely vulnerable, people end up criminalized in squats, homeless on the street, or in rare cases, “living” permanently on and off public transport for over 20 years (Menzies 2020). As I neared the end of my PhD, it became clear to me that what I had characterized as “homelessness” was in fact the domestic branch of the much bigger story of global land enclosure (theft by elites)—forced displacement. Between 2015 and 2017, as the numbers of people fleeing war in places such as Syria, Afghanistan, Iraq, and Iran were at their peak, I felt compelled to take my research in the direction of how displaced people were surviving “through things” in contemporary Europe.

Inspired by both The Ludlow Collective and the Black Trowel Collective, I sought to find people with whom to develop a Collective that would use archaeological approaches to understanding the material and visual culture of forced displacement in Europe. The Ludlow Collective used archaeology to explore the “hidden” massacre and class struggle in the coalfields of Colorado (1913–14) (The Ludlow Collective 2001), where the Black Trowel Collective (BTC) is an international group of anarchist archaeologists, each working on very different aspects of archaeology all over the world, united through their commitment to both archaeology and anarchism. Experience had taught me that if I was to study the material and visual culture of contemporary displacement in Europe with any accuracy or meaning, I had to work *with* – not *on* or *for* – displaced people. But would people currently experiencing the uncertainties and stresses of forced displacement have the time or inclination to work with me? The only way to find out was to put myself in positions where I could meet people and ask them.

## Meeting Displaced People: Greece, the UK, and Sweden

The world of contemporary forced displacement is precarious in the extreme, but I initially identified four European “hot-spots” of displacement and planned to spend time in each of them, volunteering and getting to know people. I planned to work in Greece, the UK, Sweden, and Serbia, hoping that this would give me some broad understanding of how displacement affected people in different “corners” of Europe. The Covid-19 pandemic struck before I had been able to visit Serbia; however, I was fortunately able to complete fieldwork in Athens (Greece), Plymouth (UK), and Krokom (northern Sweden) before the first UK lockdown was imposed in March 2020. Owing to the very different characteristics of each place and my relationship with them, my approach to meeting displaced people varied from country to country. For example, Athens is a city that I know well and is a place where I had strong contacts from a previous research project (Kiddey 2019). It was easy for me to meet displaced people in Athens because I had stayed in touch with many using Facebook and WhatsApp. While some people I had met in 2017 had successfully made the journey northwest and were no longer based in Athens by the time I went back (several times between 2018–19), a good number of people were still waiting for their asylum-claims to be processed, stuck living in the same displacement camps and housing squats. I flew to Athens in 2018 and called people. I was very soon back volunteering in squats and camps and in touch with people on the ground, some of whom went on to become members of The Made in Migration Collective. 

Plymouth is a city that I know very well since it is close to my home. Plymouth is a “dispersal” city, places where asylum-seekers are sent by the government to live while they wait for their asylum claim to be processed. The practice of dispersing asylum-seekers has been quite common across Europe for some time, often framed as attempting to reduce the concentration of asylum-seekers living in one place (Robinson 2003). In reality, the practice of dispersing asylum-seekers to cities where accommodation is cheapest (and often of very poor quality) is largely the result of the 2010 privatization of asylum-housing in the UK, a casualty of austerity policies, often resulting in what Jonathan Darling (2016:485) refers to as “policy-imposed-liminality.” In Plymouth, I spent time attending and volunteering at workshops, activities, and community events for asylum-seekers and refugees and came to meet people this way. I was also awarded a Public and Community Knowledge Exchange Fellowship from the Social Sciences Division of the University of Oxford, which enabled me to work collaboratively with START (Students and Refugees Together), a local refugee support group and Dr. Sana Murrani, an architect at the University of Plymouth. Sana and several displaced people whom we worked with later joined The Made in Migration Collective. 

In Athens and Plymouth, I took time to get to know displaced people, using a blend of community activist approaches and methods drawn from ethnography, anthropology, and cultural heritage studies. My hope was that varied life experiences, skills, knowledge, and efforts would combine through the development of a research collective, enabling co-interpretation of data and the translation of archaeologically sourced data in such ways that findings could be made widely accessible to diverse audiences (cf. Zimmerman et al. 2010). As I volunteered in and visited various support centers and squats, I explained that I was an academic researcher interested in documenting how “things” or “stuff” featured in experiences of displacement. What sorts of objects did people find “useful” and why? How do particular belongings connect people to the places and people left behind? How is personal and cultural identity affected by displacement and what role do material objects play in sustaining or changing identity? I spoke to literally hundreds of people and for many, it was just an interesting conversation, a way to kill time but nothing more. However, very gradually, small informal groups of people interested to continue working with me formed in each place.

I did not know northern rural Sweden at all before I visited for fieldwork in April 2019. In the next section of the article, I home in on how I came to meet displaced people using a rather unorthodox method of public engagement.

## Engelsk Fika (English Tea Party) as a Method of Community-Archaeological Public Engagement

Krokom is a very small town in Jämtland, northern Sweden. I had wanted to see for myself how displaced people were living in rural Sweden for some time because in the UK the popular view, in 2016, was that Sweden was the European country that was welcoming refugees in the best way – accepting more asylum applications and being the most hospitable (this is not a view that I share personally). I was fortunate to lodge with a wonderful local couple on their eco-small holding. Mimi worked as a support assistant at the local ‘kommune’ (integration service) with displaced people who had recently arrived in Sweden through the United Nations High Commissioner for Refugees (UNHCR). Mimi allowed me to shadow her and introduced me to scores of people from Eritrea, Sudan, Congo, Syria, Afghanistan, Iraq, Armenia, and Iran, all of whom had recently made new homes in Krokom or in tiny – no shop, no garage, no post office, *tiny* – villages, in its wide, snowy surrounds. I shadowed Mimi as she visited people to help them with issues to do with housing, documentation, and language classes, etc. Mimi introduced me as a researcher keen to work with displaced people on a project that sought to understand how people make home in new places and how material “things” are involved in that process. Most people looked utterly perplexed and often the conversation did not go any further to start with, but everyone we visited made me very welcome and offered me food and drink. It struck me that the process of “breaking bread” together has long been recognized by many cultures as a method of introduction, a way to get to know people better. Indeed, the blending of archaeological performance and eating together is not at all new (Hamilakis and Theou 2013:191–192).

With Mimi’s help, I hatched a plan to hold an *Engelsk fika* (an English tea party) in the hope that returning the hospitality might enable conversations about material culture to flourish. Mimi and I spent a day baking Devon scones and lemon drizzle cake, making cucumber sandwiches, and wondering whether it was mean to introduce people, displaced from all over the world, to Marmite (a peculiar English delicacy, usually spread on toast). Mimi made some calls and obtained permission for us to use a local church as the venue for our *fika*. We invited everyone we could think of who was new to Krokom and its surrounding villages and arranged community transport for those who lived some 40 km further north. We invited people to attend the party, to socialize and connect with others living locally, and to find out more about the material culture project. When everyone was settled with tea and cake (studiously avoiding the Marmite), I gave a very short, informal presentation in which I explained the aim of the project in simple terms, using pictures of some of the “things” and places that mean a lot to me—my kids, my dog, the beach, various notebooks, the Zippo lighter that my grandfather kept throughout his five years in prison-camp, meals I had cooked for special occasions, etc. Volunteers translated what I said real-time into Swedish, Arabic, Tigrinya, and Kurdish. Slowly at first, individual people asked me further questions. Things were clarified. A few people understood what I meant by “special objects,” “things that really matter to you,” “things that hold memories,” or “things that connect you with other people and places,” and following several hours of deeper conversation (in multiple languages), people started to show me all sorts of objects, clothing, and talismans. Tiny things were drawn from pockets, wallets, and bags, which were important and contained rich, diverse stories about people and places – the material culture of forced displacement. By the end of the day, I had received many invitations to visit people at their houses, so that they could share *their way of living* with me, in unique social and material forms.

Before I elaborate on different people’s ways of “doing” (Holloway 2010:27), I want to dive deeper into how The Made in Migration Collective evolved.

## Developing the Collective

Throughout my time volunteering and working with displaced people in a variety of places, I discussed my aim to build a research collective that would collaboratively document the material and visual culture of forced displacement in Europe and use these findings to co-curate public exhibitions with the aim of educating diverse audiences and challenging stereotypes about people who migrate. Slowly, organically, some of the displaced people with whom I washed pans, sorted clothes, and painted walls in squats and other sites of temporary accommodation expressed interest in forming a collective. Initially, the concept of working together in a non-hierarchical way was quite alien to a lot of people. It took months for one member of the Collective to stop calling me “Dr. Rachael” and just call me Rachael. But gradually, a hardcore of 12–15 people merged into The Made in Migration Collective. To my mind, all community archaeology projects could usefully benefit from explicitly seeking to form a Collective – that is, agree to principles for using a non-hierarchical structure, where individual skills and knowledge may well be useful but where there is no infallible authority and everyone is equally able to question, critique, or dismiss ideas about how to proceed (cf. Eddisford and Morgan 2018:150; see also, Angelbeck and Grier 2012).

For Nishnaabeg writer and activist Leanne Betasamosake Simpson, the process of engagement – the *how* we do things – is critical to resistance and resurgence in the face of ongoing colonialism. She describes how she engaged deeply with Nishnaabeg systems and ethical practices “including story or theory, language learning, ceremony, hunting, fishing, ricing, sugar making, medicine making, politics, and governance” (Betasamosake Simpson 2017:19). Through this deep engagement, a different understanding of resistance and resurgence emerged. She writes:

It became clear to me that *how* we live, *how* we organize, *how* we engage in the world – the process – not only frames the outcome, it is the transformation. *How* molds and then gives birth to the present. The *how* changes us. *How* is the theoretical intervention (Betasamosake Simpson 2017:19, emphases in original).

There are similarities between this Nishnaabeg ethical practice and system of relations and that of the anarchist notion of prefiguration. The term “prefiguration” was originally coined over 40 years ago, by Carl Boggs (1977) but re-emerged more profoundly within archaeological and anthropological literature particularly in connection with David Graeber’s (2013) influential work. For Lewis Borck and Matthew Sanger (2017:9), prefiguration comes from the anarchist idea that:

societies are prefigured, which is to say they emerge from the practices that create them. Instead of the ends justifying the means, anarchists believe that the means create the ends. But also, that the means, in some way, are the ends. The two are simultaneous. The ends are process.

Luke Yates (2015:13) defines prefiguration as a “necessarily plural configuration of practices” in which every action is perceived to contribute to “building capacity” and “achieving distant future political goals.” Similarly, in their analysis of the UK based intersectional feminist anti-austerity group, Sisters Uncut, Armine Ishkanian and Anita Peña Saavedra coined the term “intersectional prefiguration,” which they contend enables us to understand how social movements challenge existing power relations, not just by focusing on what the organization achieves but by acknowledging and addressing inequalities *within* the organization which can affect group cohesion and the ability of a social movement to achieve its wider aims (Ishkanian and Pena Saavedra 2019:986). Ishkanian and Peña Saavedra (2019:986) maintain that:

enacting intersectional prefiguration is predicated on actors developing a collective identity, embracing a commitment to organizing intersectionally and adopting specific organizational methodologies through which to put it into practice. In the practice of intersectional prefiguration, means and ends are not separate and changes at the level of policy and society are seen as indivisibly connected to the changes at the individual and organizational levels. (emphasis in original).

This point about developing a collective identity was crucial to the formation of The Made in Migration Collective* .* As opposed to seeking to work collaboratively with a marginalized or “imagined” (Anderson 1983) community based on social identity, The Made in Migration Collective represents the *creation of a community with a diverse identity* based around an archaeological subject – the materiality of contemporary forced displacement in Europe. The Collective welcomes anyone who is willing to cooperate using the broadly anarchist notion of prefiguration and consensus decision making principles (explored in more detail below). The types of contribution that members of the Collective make to the project vary. While most members have personal lived experience of displacement in Europe, three of us have no personal experience of forced displacement but contribute the knowledge and skills that allow is to collectively record and reflect upon the material and visual culture under study (for example, one of our members is an activist filmmaker, another is a professional poet, another an architect). But our collective identity as The Made in Migration Collective and our commitment to intersectionality enables us to transcend our not insignificant differences in pursuit of our shared aims. For example, we are of mixed gender, and mixed social, ethnic, and cultural backgrounds. We are intergenerational, ranging from age 18 to 73 years old. Members of the Collective are of mixed sexual orientation, educational backgrounds, and personal political leanings.

Consensus is used to make decisions within The Made in Migration Collective. We use a similar consensus reaching process to that used by the Black Trowel Collective (Morgan 2021a). All members of The Made in Migration Collective have four options as to how they respond to a particular idea. Everyone can choose to: (1) vote yes, (2) vote no, (3) stand aside (choose not to be involved in the decision due to ambivalence or a conflict of interests), or (4) block. The option to “block” differs from “vote no” because to “block” means that the idea cannot proceed, even if everyone else “votes yes.” Equally, a “block” vote can be contested. Discussions conducted using such a process are time-consuming at best, left unresolved at worst. However, by prioritizing group consensus over, for example, a tight timeline of multiple outputs, the Collective learns, produces, and moves *together*. We move forward together despite our not insignificant differences (and sometimes we move backward or stall together, but we don’t move until we have reached a point where everyone is happyish with the direction of travel). We work at the pace that is right for the Collective as a whole and this results in learning in all directions.

It is important to acknowledge that for all my attempts (and those of many others) to make the world a fairer place, it is not a fair place. Even while adopting a non-hierarchical model for working together and forming a Collective using prefiguration, power relations between displaced people and researchers are all too real. All displaced people are vulnerable from the moment that they cross a border without the “correct” documentation by dint of the precarious legal statuses that nation states ascribe to them. Displaced people often also find themselves in dependent and unequal relationships with humanitarian aid providers, officials, and researchers. In light of this, it was important to be extra sensitive to inherent power relations between the people with whom I sought to work – displaced, often facing unimaginable precarity – and myself –a Brit with a passport and a home to go to. This tension remains a source of constant reflection for me. For my part, I was extremely careful to make communications very clear and to ensure ample opportunities for people to discuss and question the proposed project or a particular aspect of our work. I made sure that everyone who got involved knew that they could leave the project at any time, without having to give any reason. I stressed that the Collective could not change or improve people’s immigration status, that people joined because they found the prospect interesting.

Non-hierarchical systems of social relations are not always easy. Disagreements do, of course, occur and working through these to find a solution with which everyone is happy(ish) takes a lot of time and effort, as it should if consensus is truly prioritized (for example, see Casella and Piprani, this volume, for their description of how a row erupted in a collective housing project over how to pay for a chicken coop). My friend and colleague Alfredo González-Ruibal has long criticized my work for appearing to uncritically “celebrate” all that is “popular, bottom-up, and local” (González-Ruibal 2018a:509), but my work is not always “fluffy” (González-Ruibal 2018b). The Made in Migration Collective has certainly suffered tough times, not least when three members of the Collective (two originally from Armenia and one from Azerbaijan) instantly fell out when the ongoing feud between those countries flared into war again on September 27, 2020. This coincided with a series of online interpretation workshops during which members of the Collective met on Zoom to co-interpret and curate material for our virtual exhibition. Our three colleagues – two based in northern Sweden and one based in southwest England at that time, approximately 1,700 mi (2,736 km) physically apart from each other – could not face one another on Zoom despite having worked together weekly for months. As the war raged for six weeks, our colleagues became sworn enemies. For everyone’s mental well-being and safety, I intervened. I suggested to the Collective that we did not discuss war or conflict in any capacity, and instead turned our attention to the practical elements of co-producing a virtual exhibition. The Collective agreed unanimously. I continued to offer support individually to those directly affected by the Nagorno-Karabakh war. However, our two Armenian colleagues decided to leave the project. I offered to put them in touch with trauma counseling and psychological support services local to them in Sweden and we had several very long conversations about how they wished the project well but the prospect of working alongside someone from Azerbaijan was too much. Although it pales into insignificance compared with the triggering effect that reports and images of war on news bulletins and social media feeds have on my displaced colleagues (all of whom fled war), I was very disturbed by the hatred espoused by all parties. It radicalized me further against nationalism and borders – imaginary lines drawn on our shared planet which are hardened and heavily policed and which fuel bloody destruction of people and places.

## Three Cracks in Capitalism from The Made in Migration Collective

### Senbetu’s *jebena*, Krokom, Sweden

An Eritrean woman, Senbetu, now living in Sweden, invited me to enjoy coffee with her and her family at their home, prepared as it is traditionally in Eritrea, a ritual that takes roughly two capitalist hours (Lukàcs 1971; Simmel 2004). With her then 17-year old daughter acting as our interpreter, Senbetu told me:

It is impossible to tell you how I am Eritrean without the coffee! The importance of making coffee this way to Eritreans…gah! First, I roast the beans and toss them on a plate…the aroma passes around the family and guests for a long time…it is the way that we socialize. Come!

How could I claim to collaboratively document the role of material culture in an Eritrean woman‘s lived experience of forced displacement by just photographing, drawing, and measuring her family *jebena* (coffee jug)? What would I learn other than the style and size of a random coffee pot? The *jebena* is not just a coffee pot. It is Senbetu’s *jebena* and it relates to Senbetu’s memories of people and place, and it is partially obscured if it is not socially active (Appadurai 1986). This is basic ethnographic participant observation, but it is more than that. It is about respecting the value and meaning of objects to the people to whom they belong and respecting the time and space that using these objects traditionally take up. I interpret this in much the same way that Eric Drake (this volume) interprets the labor of a nineteenth-century Anishinaabe logging family “doing-in-against-and-beyond” the logic and relations of capital as part of their struggle to survive at a time (ca. 1840–1940) when Native American communities and social practices were the target of federal Indian assimilation programs and subject to the disruptive forces of industrial capitalism. In a similar way, Senbetu’s coffee making ritual is a way for her, her family, and their friends in Sweden to live their own “good” life, by performing a conscious activity that serves community building. As Attila Dézsi and LouAnn Wurst (this volume) note in their introductory article, Senbetu is engaged in what Holloway would term “doing” (Holloway 2010:27), where he distinguishes “doing” from “work” as concrete labor, while retaining the term “labor” for abstract production for exchange value. As Holloway (and Dézsi and Wurst) reminds us “the central issue is not the terms we use, but the distinction between the two aspects of human activity and the relationship between them... there is a constant living antagonism between abstract labour and concrete doing” (Holloway 2010:517–518).

**Fig. 1:**
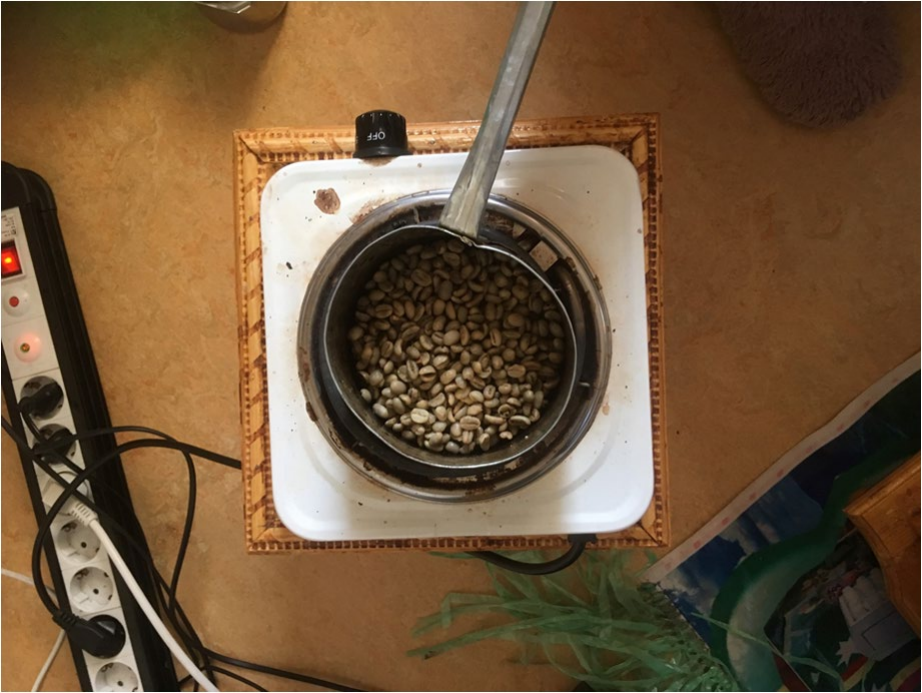
Making coffee, Eritrean style (Sweden 2019)

A few days after the *Engelsk Fika*, I made my way to Senbetu’s house, where I was greeted with hugs and kisses. Senbetu’s daughter translated from Tigrinya and Swedish, into English. Several younger siblings clambered on me, showing me toys and inviting me to play. An older brother, wearing a pinafore, helped his mother to prepare food. I had thought I was going for “coffee,” but it soon emerged that coffee was just one part of the hospitality. Once the food was ready, I was invited to eat first, with the whole family watching me. This initially felt very awkward to me but the gesturing from Senbetu and the anticipation on the faces of her family required no translation. I tucked into delicious plates of chicken and spiced vegetables. After dinner, we moved to the sitting room where Senbetu produced a beautiful *himbasha* (Eritrean flatbread, made for special occasions). She carefully pulled out a small electric stove and a saucepan, gesturing to me to take photographs of each step of the process (Fig. 1). As I sat on the sofa, Senbetu oscillated between rhythmically swirling the coffee beans in the pan, roasting them until they were just the right color, releasing a delicious smell, and directing her daughter to show me more “things.” It took two hours for coffee to be prepared traditionally – “it’s the way we socialize!” – so, we chatted. Senbetu’s daughter showed me photographs of the family left behind in Eritrea, some dating to the early 1980s. Senbetu’s husband, who had been friendly but silent for the whole of my visit so far, commented that what he missed most about his home, aside from his family, was his goats and having both the outside space and local horticultural knowledge to grow vegetables. This afternoon, this ritual, this family’s welcoming social space, was a big fat crack in capitalism’s face! In making coffee according to her own tradition, taking the time and space to “do” in ways that felt right for her, Senbetu beamed, the result of her own labor (Marx 1990 [1867]). Sayers makes this point better: “Through work, the worker relates not only to the object of work and hence to the natural world, but also – and through it – to other human beings” (Sayers 2003:108).

### Scrap Co-Op, Athens, Greece

**Fig. 2:**
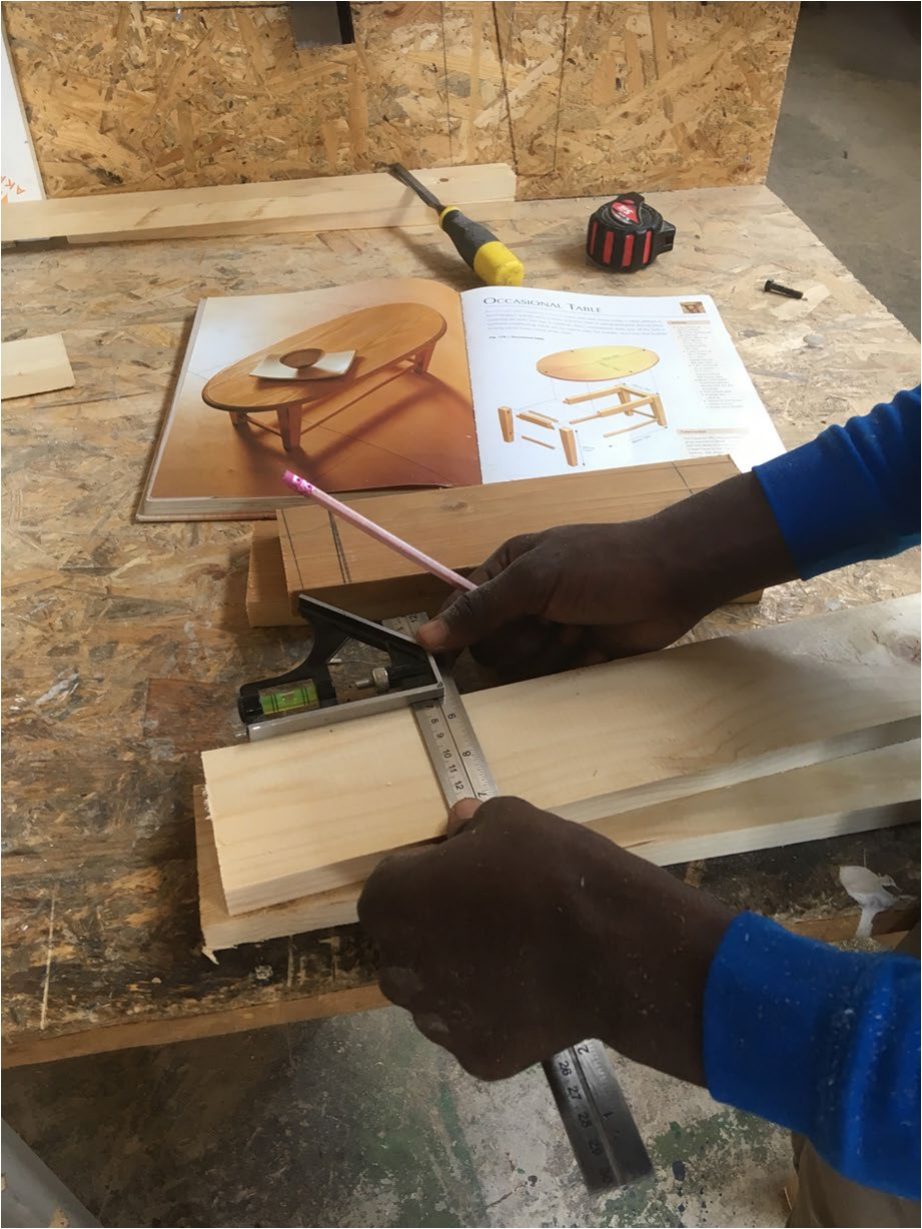
A man making a table at Scrap Co-Op, Athens, 2019

The one thing that displaced people have is time – endless sprawling time—as they wait indefinitely for months and in some cases, years for asylum claims to be to be processed (cf. Dawdy 2010). If viewed as unintentional “unemployment,” the time that displaced people spend waiting could be seen as a capitalist crack (Dézsi and Wurst, this volume). Most of the people I have conducted collaborative fieldwork with during this project want to work but are prohibited by nation states from doing so. In Athens, many people have taken (illegal) casual work as construction workers only to return from a ten-hour day in the hot Greek sun with less than €15 pay. Of course, they are exploited. To whom could they complain? However, also in Athens, there are examples of “sprouts” being tended through community projects that explicitly set out to provide displaced people with the opportunity, training, materials, and space to learn new or practice and teach familiar skills. The Scrap Co-Op is one such place (Fig. 2).

Scrap Co-Op grew out of a workshop space which had been part of a different Athens-based co-operative (Khora). Unlike most co-operatives and NGO projects intended to provide support and services for displaced people living in Athens, Scrap Co-Op is based much further out of the city center, close to the UNHCR camp for displaced people, Eleonas. At Scrap Co-Op, there are lots of tools and woodworking materials and they also run electrician courses so that people can become certified. Upstairs, there is a jewelry making workshop. The Scrap Co-Op is open between 11am and 7 pm every day and several people with whom I spoke in the camp told me that they spend these hours at Scrap Co-Op because it is a much nicer place to be. On the ground floor there is an airy, friendly reception area with information about the types of courses that are available. The language courses – when I last visited in 2019, English and Greek were offered – are totally booked up, there is such demand as people have started to realize that they are stuck in Greece, with no way forward to the countries that they initially tried to reach – Germany, U.K., Sweden – and no viable way back. Many people are now trying to learn Greek so that they stand a better chance of being able to find employment.

The founder of the Scrap Co-Op told me:

I started the project after working with lots of skilled people who were in a bad financial position but working every day for free. So, we opened a workshop where they can access the tools to start small business ventures and be *supported in doing*…it grew to teaching people as well and one of our students just got his first full time carpentry job and more are following close behind. The space can also be used to build for the other projects and squats [in Athens] and two playgrounds have been built in the space so far (my emphasis).

I spoke with a man “doing” at the Scrap Co-Op. He was from West Africa but wished to remain anonymous. He told me:

Scrap Co-Op is everything to me. I am here every day, making things that I find in books or on the internet. I feel better when I am here, making these things.

This is useful labor (Marx 1990 [1867]) – people feel better about the fact that they are working for themselves, not wasting their lives doing nothing but waiting for authorities to process or reject their asylum-claim. People who attend Scrap Co-Op are shielded from the constant pressure of capitalism, the constant reminder that they have no money – it offers people some dignity (cf. Holloway 2010:20).

### Pampiraiki Warehouse, Athens, Greece

we have more than enough to supply all the wants of the people if we could only get it distributed…If there is enough to waste, why fret for fear some one will get a little more than he gives? (Presley and Crispin  2005 [1908]:62)

Pamperaiki warehouse is a volunteer-led operation. About an hour by bus from central Athens, it was originally built as the 2012 Olympic basketball stadium – 3,500 m^2^ with a spectator capacity of 15,000. When I last volunteered there in 2019, the space was being used to process donations sent from across Europe. Clothes, shoes, wheelchairs, dry food, toys, and sanitary products were distributed among displaced people across the squats in Athens and driven in private vehicles out to the Greek Islands – all by volunteers. Previously, in 2017, the space had been full of people desperately attempting to survive in tents, with extremely minimal bathroom facilities.

**Fig. 3:**
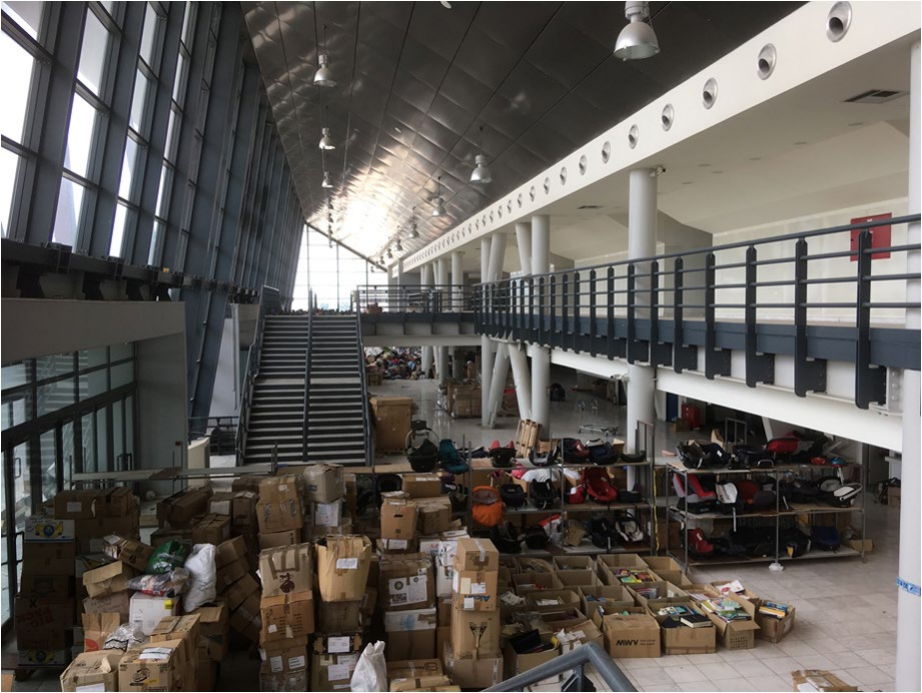
Pampiraiki warehouse, Elliniko, Athens (June 2019)

**Fig. 4:**
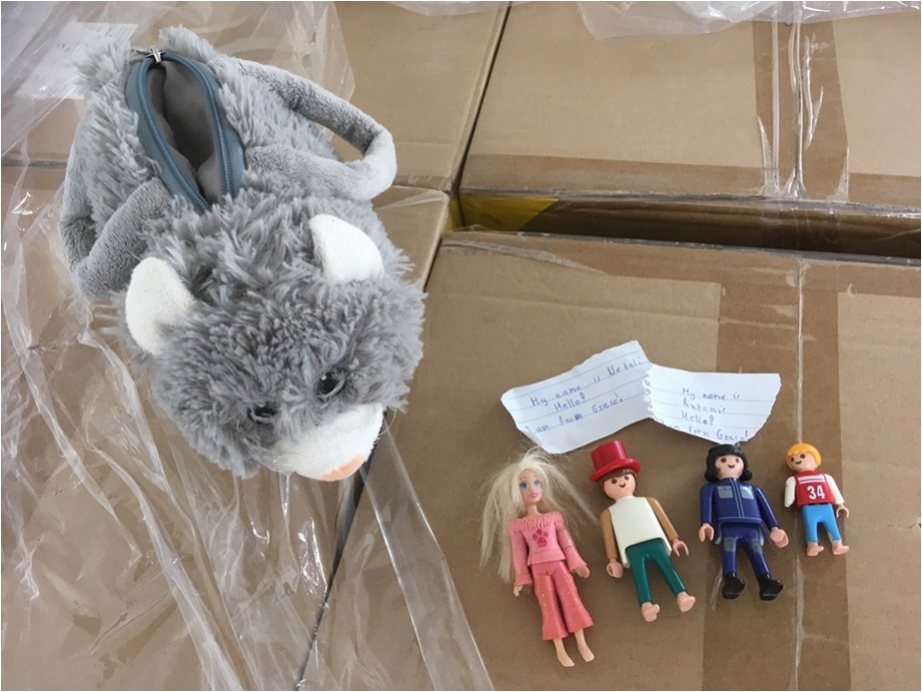
Children's toys and notes, Pampiraiki warehouse, Athens, 2019

The warehouse was a site where the extreme overconsumption and the vast levels of inequality in some parts of Europe were stomach churningly apparent (Fig. 3). Thousands upon thousands of boxes, bags, crates and pallets were stuffed full of clothes and shoes that people across Europe who had seen the news and had been motivated to donate to “refugees.” Some were very poor quality or just unhelpful – odd shoes, broken or dirty things that could not be given away. Some were just weird and made you wonder what people were thinking – a bee-keepers’ suit, a wedding dress, a single ice-skate. Some were high quality, unworn, with labels such as Levi, Sisley, Reiss, and Chanel still attached. A fellow volunteer turned to me and we briefly discussed how we each had visceral reactions to the mounds of discarded clothes – the millions of Euros and level of environmental damage involved in sourcing materials, making, shipping, marketing, advertising, buying, and donating the stuff around the world. How, juxtaposed with the poverty in the world, it was genuinely sickening. “I don’t know whether to be angry or just sad,” said my interlocutor. We agreed that, if everyone were to see this, no one in their right mind would ever buy new clothes again. In an act of “joyful mourning” (Morgan, 2021b:3) familiar to anarchists and activists, my interlocutor and I spent an hour at the end of our shift rifling through boxes for specific items requested for the Khora Free Shop which provides donated clothes and sanitary items to people who need them in Athens. At the time, these were men’s T-shirts, new underwear and socks, baby things, children’s clothes, and summer clothes appropriate for Muslim women. I found a child’s bag, shaped like a cat. Inside were four small toys and notes, presumably from the children who had donated them – a tiny speck of hope for the future of humanity in this warehouse of waste and goodwill (Fig. 4).

At the time I was there, the warehouse building was owned by the Greek state but Pampiraiki had been served an eviction notice and the building had been sold off to developers for a “luxury complex.” A comment in a 2021 press release quotes the Greek finance minister:

[the site] will be a metropolitan pole of global appeal which… will showcase Athens as a tourist destination, a business hub and a spot of recreation in the wider Eastern Mediterranean region. Finance Minister, Christos Staikouras, cited in (Reuters 2021).

As James C. Scott (1985:39) put it:

By creating and disseminating a universe of discourse and the concepts to go with it, by defining standards of what is true, beautiful, moral, fair, and legitimate, [the elites] build a symbolic climate that prevents subordinate classes from thinking their way free.

We lost this crack but those involved in the Pampiraiki warehouse have not given up on thinking of ways to be free. By documenting the creation and destruction of frontline alternative spaces such as Pampiraiki warehouse, we will inevitably be tossed about in a sea of raging emotions but if we hold fast to our proven methods, collaborative contemporary archaeology has an important role to play in providing robust evidence for how capitalism reproduces its violence and contributing usefully to the wider discourse on, and active calls for, degrowth (Kallis 2018).

## Conclusion

It seems an age since the Society for Historical Archaeology’s annual conference in Boston, January 2020, when I stood outside the hotel with LouAnn Wurst discussing John Holloway’s book, *Change the World Without Taking Power* (Holloway 2010). We had no idea that the Covid-19 pandemic was already underway thousands of miles across the planet. I had barely touched down in London from my last stint of fieldwork in Athens (December 2019) when I was back on a plane to the US – that level of international travel seems unethical to me now. Internally, I was struggling with the juxtaposition of what I had left in Athens – hunger, cold, uncertainty, trauma—and the shiny surfaces and expensive drinks at the Sheraton, Boston. It occurred to me more than once that I was wasting my time playing at academia and I should turn my attention to more radical activism. Then the Covid-19 pandemic effectively grounded me at my comfortable home in south Devon (UK) and everyone’s plans fell apart.

During those first few months of the pandemic, I stayed in touch with as many members of The Made in Migration Collective as I could using WhatsApp. We decided to continue working together through a series of online workshops. Sadly, this meant that several of the people I had been working with in Athens fell away from the project, not having regular access to the internet or computers. Zoom is hard enough—it is even more difficult if you share an internet enabled device with several other people and the device is a phone with a cracked screen and the only available internet is in a public area. But 12 members of The Made in Migration Collective spent a lot of time between April 2020 and June 2021 meeting online regularly to discuss and co-interpret data. Not being able to bring specific people and artifacts physically together for a live public heritage exhibition as we had originally intended, we set to work playfully reflecting on those objects and belongings, using a mishmash of mixed methods from filmmaking to creative writing to memory mapping and recording audio over still photographs. Through memory work, our “network of voices” started to sing. We co-presented our works in progress at the online festival CHAT in November 2020 (The Made in Migration Collective 2020) and launched a co-curated virtual exhibition called *“Made in Migration”* during Refugee Week 2021 (https://rachaelkiddey.co.uk).

**Fig. 5:**
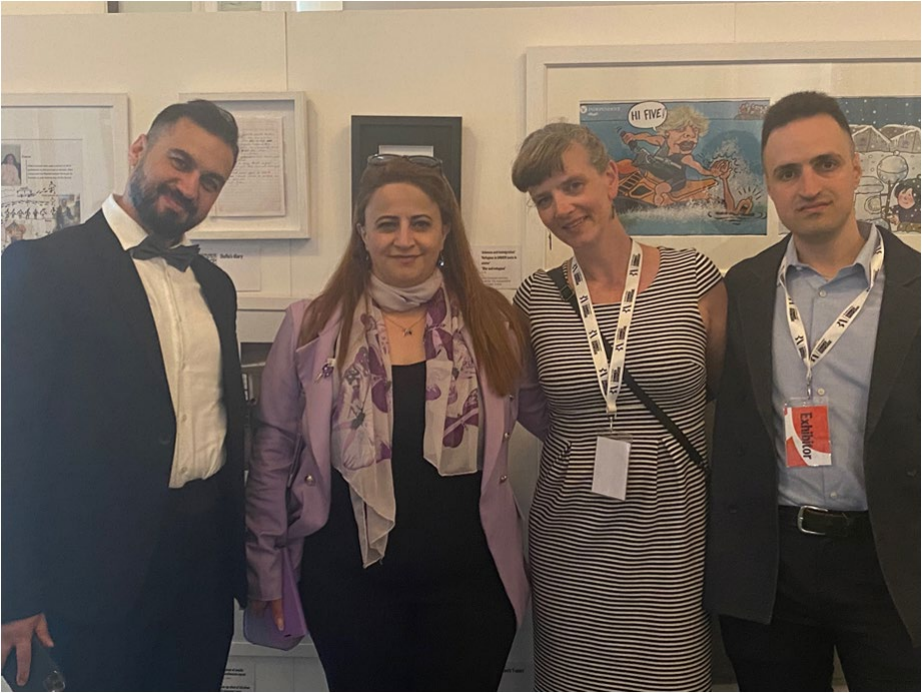
Some of The Made in Migration Collective members, London, 2022

Almost a year to the day after the launch of our virtual exhibition, we were awarded British Academy funding to take a “live” version of the digital show—*Made in Migration, IRL (In Real Life)*—to the heart of London, where it featured as one of 12 exhibits in the British Academy Summer Showcase 2022 (Fig. 5). Two members of the Collective – Hassan and Alaa – came from Sweden and Gunel traveled to London from Plymouth. The four of us spent three happy but exhausting days talking about our work with visitors, including school children, university students, academics, MPs and top civil servants, charity CEOs and business owners. Watching my smartly dressed colleagues explain eloquently to a retired Conservative MP why they had crossed borders “illegally” was perhaps our greatest “crack” so far.

As a Collective, we have been busy and while one part of our project is now complete, we are taking a short break before coming back together to do more “doing” (Holloway 2010:27). Nothing is decided yet but on the table are ideas for a documentary film, a children’s book about displacement, and a peer-reviewed cartoon series. What is certain is that, as a Collective that uses consensus-based decision making, we have a truly diverse identity; we are building capacity and focused on achieving some “political” goals.

Part of what we tried to do with *Made in Migration* – the virtual exhibition and the live exhibit—was to document, collect and collate the very few remaining, often small, utilitarian material belongings that displaced people had been able to keep with them through their journeys, and to use mapping techniques to illustrate the messy and uncertain journeys that displaced people must navigate. We wanted to demonstrate, through material and visual culture, the practical concerns for people “on the move” – how people kept safe and in communication with loved ones—and the myriad personal ways in which “things” function as mnemonic memorials to places, people and things lost to displacement (cf. Theune 2018). Material things are essential to human life, not just necessary for biological survival (for example, food, medicine, shelter, clothing etc.) but they are equally vital to psychological and ontological survival, necessary for sustaining social bonds and human relationships (cf. Olsen 2010). I will explore the objects, artifacts, maps, films, and poems which form the content of our two public exhibitions in more detail in a forthcoming book.

Nationalism is “fissured with the politics of power, rights and inauthenticity” (Tolia-Kelly 2019:591). Archaeology has an important role to play in educating diverse audiences about myriad ways to be – or to have been – authentically human, by documenting how human beings adapted – or adapt – the material and natural world. The Made in Migration Collective focused on lived experiences of forced displacement but the model would work just as well for documenting lived experiences of skateboarders or anyone else. As archaeologists, it is our role to facilitate investigation into and the co-production of knowledge about how humans relate to the material and natural world throughout time and across the planet. Some of those archaeologies (always plural) will document aspects of nation-building but many more will document extraordinarily ordinary experiences that may seem alien to those who have not lived them – this is the kaleidoscope that is humanity! It is not the role of archaeologists to only produce narratives of the past or historical present which are sanctioned by those with a nationalist agenda although archaeology is sometimes used in this way (see Mizoguchi and Smith 2019). If we are content with nation states dictating which pasts are “allowed” to be known, we will continue to suffer from racism and increased division because it does not suit nationalist ideologies (or economies) to entertain “alternative discourses that renounce the establishment of hierarchies and emphasize the continuity and connections that should prevail between humans and non-human nature” (Estévez-Saá and Lorenzo-Modia 2018:129) – and material culture.

I am not suggesting that we storm the Houses of Parliament – although, as we saw in January 2021 at the Capitol in Washington DC this is a very real threat in the face of advanced racism and alienation. I am instead imploring that we – the 99% of us who are not billionaires or presidents (cf. Solnit 2011) – adopt prefigurative approaches to enhance our personal and professional connections. Between us, the millions of ordinary people of the world, we must seek ways to understand our neighbors better and not through the toxic lenses of nationalism, capitalism, and the corporate media. Community archaeology is a good model for these types of conversations. Community archaeology enables multidirectional, multisited, intercultural learning, and the Internet enables us to link up our pro-peace, pro-equality groups. I leave the last word to John Holloway, whose book helped me to recognise that the “scream” I felt as I left Syntagma station was not the beginning of a break-down, but a perfectly rational response to witnessing the “horrors of capitalism” (Holloway 2010:9): “For what is at issue in the revolutionary transformation of the world is not *whose* power but the very existence of power. What is at issue is not *who* exercises power, but how to create a world based on the mutual recognition of human dignity, on the formation of social relations which are not power relations” (Holloway 2010:17–18).
